# Protein kinase C controls activation of the DNA integrity checkpoint

**DOI:** 10.1093/nar/gku373

**Published:** 2014-05-03

**Authors:** María Soriano-Carot, Inma Quilis, M. Carmen Bañó, J. Carlos Igual

**Affiliations:** Departament de Bioquímica i Biologia Molecular. Universitat de València, 46100 Burjassot (Valencia), Spain

## Abstract

The protein kinase C (PKC) superfamily plays key regulatory roles in numerous cellular processes. *Saccharomyces cerevisiae* contains a single PKC, Pkc1, whose main function is cell wall integrity maintenance. In this work, we connect the Pkc1 protein to the maintenance of genome integrity in response to genotoxic stresses. Pkc1 and its kinase activity are necessary for the phosphorylation of checkpoint kinase Rad53, histone H2A and Xrs2 protein after deoxyribonucleic acid (DNA) damage, indicating that Pkc1 is required for activation of checkpoint kinases Mec1 and Tel1. Furthermore, Pkc1 electrophoretic mobility is delayed after inducing DNA damage, which reflects that Pkc1 is post-translationally modified. This modification is a phosphorylation event mediated by Tel1. The expression of different mammalian PKC isoforms at the endogenous level in yeast *pkc1* mutant cells revealed that PKCδ is able to activate the DNA integrity checkpoint. Finally, downregulation of PKCδ activity in HeLa cells caused a defective activation of checkpoint kinase Chk2 when DNA damage was induced. Our results indicate that the control of the DNA integrity checkpoint by PKC is a mechanism conserved from yeast to humans.

## INTRODUCTION

Genome integrity maintenance is a major concern for cellular physiology. Genetic material is constantly exposed to damage. Indeed, it has been estimated that every single cell in the human body is subjected to several thousands of deoxyribonucleic acid (DNA) lesions every day (1,2). Some of those aberrations are due to physiological processes, such as replicative errors, deficient activity of some enzymes or reactive oxygen species. DNA damage is also produced by external physical and chemical sources. To counteract such threats, cells have evolved a mechanism that detects damage, transduces the signal and triggers an accurate cellular response to maintain genome stability. This surveillance mechanism is known as the DNA integrity checkpoint (3,4). Proper checkpoint functioning is crucial for cell viability and for preventing diseases like cancer.

The DNA integrity checkpoint is highly conserved from yeast to humans. Major regulators of the response to DNA damage in *Saccharomyces cerevisiae* are Mec1 and Tel1 kinases, ATR and ATM, respectively, in mammals (5–8). Both kinases collaborate in the maintenance of genome integrity responding to different types of DNA lesions. Mec1, in a heterodimeric complex with the Ddc2 protein, senses stretches of single-strand DNA (ssDNA) coated by ssDNA binding protein RPA, which are generated when a wide variety of primary lesions, including nucleotide or base alterations, crosslinks or double-strand breaks (DSBs), are processed and when replicative forks are stalled (9). ssDNA-RPA is also recognized by the clamp 9-1-1 complex and loading clamp complex Rad24–RFC, which mediate Mec1 activation (10–14). Meanwhile, Tel1 senses the blunt ends of unprocessed DSBs (15), which are also bound by the MRX complex (Mre11–Rad50–Xrs2). Having sensed damage and activated Mec1 and Tel1, a series of phosphorylation events affecting Ddc2, the Ddc1 clamp protein, Xrs2 and histone H2A and the participation of adaptor protein Rad9 (or Mrc1 in replicative forks) leads to the recruitment of checkpoint effector kinases Chk1 and Rad53 (Chk2 in mammals), which are activated by Mec1 and Tel1 (16). Rad53 mediates most of the response in budding yeast cells. After being activated, Rad53 is released from chromatin to act on critical targets that promote cell cycle arrest (17–20). Additionally, Rad53 targets factors to induce the expression of DNA repair genes (21), stimulates ribonucleotide reductase activity (22,23), suppresses replication origins firing and stabilizes replication forks (24–28). In the case of pluricellular organisms like humans, a key mediator of the cellular response is tumor suppressor p53 protein, which is activated by kinases ATR/ATM and Chk1/Chk2. If severe damage is beyond repair, p53 induces permanent cell cycle arrest (29,30) and apoptosis (31).

In eukaryotic cells, protein kinase C (PKC) plays a crucial role in the regulation of growth, proliferation and differentiation in response to extracellular signals. In mammals, there are at least 12 genes coding for different PKC isoforms (32–34). The regulatory domains present in PKCs include the C1, C2 and HR1 domains, which mediate regulation by DAG, Ca^2+^ or Rho GTPases, respectively. Based on the presence and functionality of these regulatory domains, mammalian PKCs are divided into conventional PKCs (PKCα, PKCβ, PKCγ), novel PKCs (PKCδ, PKCε, PKCη, PKCθ), atypical PKCs (PKCι, PKCζ) and PKC-related kinases (PRK1, PRK2, PRK3). *S. cerevisiae* contains a single PKC, Pkc1, which can be considered an archetypal PKC as it contains all the regulatory domains, although C1 and C2 are non-functional. In *S. cerevisiae*, the PKC pathway is essential and mediates the response to environmental stresses and morphogenetic processes that affect cell surface (35–37). Pkc1 is activated by GTPase Rho1 after cell wall stress detected by membrane-sensor proteins. Pkc1 in turn phosphorylates and activates an mitogen-activated protein kinase (MAPK) module constituted by the mitogen-activated protein (MAP) kinase kinase kinase Bck1, the redundant pair of MAP kinase kinases Mkk1 and Mkk2 and the MAP kinase Slt2, which ultimately induces the expression of genes involved in cell wall biogenesis.

Although the best described function of Pkc1 is the maintenance of cell integrity, data in the bibliography indicate that the PKC pathway may be related to DNA metabolism. Our group has reported a functional connection of members of the PKC pathway with replication cyclin Clb5 and with different checkpoint proteins (38). More recently, we described a connection between Slt2 MAPK and the cellular response to genotoxic stresses (39). In fact, checkpoint kinases Mec1 and Rad53 regulate Slt2 transcriptional activity in response to caffeine (40). Furthermore, the *pkc1* mutant exhibits a hyper-recombination phenotype like mutants of genes involved in DNA metabolism (41) and it is hypersensitive to genotoxic agents such as hydroxyurea (HU) (38), bleomycin (42), methyl metanosulfonate (MMS) and 4-nitroquinoline 1-oxide (4NQO) (43). Also, the PKC pathway has been related to nucleotide biosynthesis (44,45). Pkc1 belongs to the AGC (protein kinase A/protein kinase G/PKC) kinase family and PKA cooperates with the DNA damage checkpoint to restrain mitotic progression following DNA damage (46,47). All these observations led us to conduct an in-depth study on the relation of Pkc1 with cellular response to DNA damage. Our results directly link PKC in both yeast and humans with DNA integrity checkpoint activation in response to damage.

## MATERIALS AND METHODS

### Yeast strains and growth conditions

The yeast strains used in this study are shown in Table 1. The *PKC1-GFP-kanMX6* cassette was amplified from pFA6a series plasmids (a gift from Dr J.R. Pringle) and integrated in the indicated parental strain. The substitution of the *PKC1* promoter by the *tetO_7_* promoter was obtained by integrating a DNA fragment amplified from plasmid pCM225 (a gift from Dr E. Herrero). The *tel1::kanMX6* cassette was amplified from the *tel1* Euroscarf yeast strain and introduced in the indicated strains.

**Table 1.** Yeast strains

W303-1a	*MATa ade2-1 trp1-1 leu2-3,112 his3-11,15 ura3-52 can1 rad5-535*
JC6-3a	*MATa pkc1-8 ade2-1 trp1-1 leu2-3,112 his3-11,15 ura3-52 can1 met^−^*
JKM139^a^	*MATa ade1-100 trp1-1 leu2-3,112 lys5 ura3-52 trp1::hisG hoΔ hml::ADE1 hmr::ADE1 ade3::GAL1-HO*
GPY1115^b^	*MATa PKC1::HIS3 leu2-3,112 ura3-52 his3-Δ200 trp1-Δ901 ade2101 suc2-Δ9*
N1-3a^c^	*mec1-1* in W303-1a
JCY1039^d^	*mec1::TRP1 sml1::HIS3* in W303-1a
JCY1258	*tel1:: kanMX6* in *W303-1a*
JCY1271	*tel1:: kanMX6* in JC6-3a
JCY1275	*tel1:: kanMX6* in JCY1039
JCY1352	*mec1::TRP1 sml1::HIS3* in JC6-3a
JCY1471	*tTR'::LEU2 tetO_7_::PKC1-kanMX4* in W303-1a
JCY1511	*PKC1-GFP-kanMX4* in W303-1a
JCY1514	*PKC1-GFP-kanMX4* in JKM139
JCY1580	*PKC1-GFP-kanMX4* in JCY1039
JCY1583	*PKC1-GFP::TRP1* in JCY1258
JCY1654	*tel1::kanMX6* in JKM139

^a^From Dr J.E. Haber.

^b^From Dr G. Paravicini.

^c^From Dr N.F. Lowndes.

^d^From Dr J. Torres.

Cells were grown on standard yeast extract-peptone-dextrose (YPD) or YPD medium supplemented with 1 M sorbitol where indicated. For induction of genotoxic stress in liquid cultures, 0.2 M HU, 0.04% or 0.02% MMS or 5 μg/ml phleomycin was added to exponentially growing cultures or cells were exposed to ultraviolet (UV) irradiation (50 J/m^2^). For induction of a single DSB, the *GAL1:HO* strain was grown overnight on yeast extract-peptone-2% raffinose medium, then 2% galactose (or 2% glucose as a negative control) was added to the medium and cells were incubated for 4 h. Synchronization of cells in G1 or G2/M phase was obtained by incubation for 3 h in the presence of 2.5 μg/ml α-factor or 10 μg/ml nocodazole, respectively. To repress the *tetO_7_* promoter, doxycycline was added to a final concentration of 5 μg/ml.

### Plasmids

Centromeric plasmid pPKC1 was constructed by cloning a DNA fragment expanding the promoter and coding region of the *PKC1* gene (from −556 to +3925) obtained by polymerase chain reaction (PCR) amplification with oligonucleotide bearing an SphI restriction site in SphI-cleaved YCplac33. Plasmid pPKC1^K853R^ was obtained from pPKC1 by site-directed mutagenesis of the Pkc1 kinase domain using the QuikChange® Site-Directed Mutagenesis Kit (Agilent Technologies). pPKC1_p_ plasmid was constructed in a two-step process. First, the *ADH1* terminator including the stop codon (+772, +1011), amplified from pFA6a-GFP(S65T) with oligos containing a SalI or PstI site, was cloned in SalI–PstI-digested YCplac33. Next, *PKC1* promoter (−600, −1) amplified from genomic DNA with oligos containing the EcoRI and KpnI restriction sites was introduced by EcoRI–KpnI digestion. The pPKCα, pPKCε, pPKCδ, pPKCη, pPKCι and pPRK2 plasmids were constructed by cloning in KpnI–SalI-cleaved pPKC1_p_ a DNA fragment expanding the coding region of the referred isoform obtained by PCR amplification from mouse complementary DNA (cDNA) (kindly provided by Dr Isabel Fariñas) using a forward oligo containing a KpnI restriction site and a reverse oligo containing a SalI site. The pPKC1-GFP plasmid was obtained by integrating the green florescence protein (GFP) coding region amplified from pFA6a-GFP(S65T) at the C-terminal end of *PKC1* in the pPKC1 plasmid. Plasmid pXrs2-HA was kindly provided by Dr J. Torres.

### Growth conditions and transfection of human cells

HeLa cells were grown under 5% CO_2_ in Dulbecco's modified Eagle's medium supplemented with 10% fetal calf serum, 100 units/ml penicillin, 100 μg/ml streptomycin and L-glutamine at 2 mM. For DNA damage experiments, the cells were seeded out in 6-well plates at a density of 2 x 10^5^ cells/well and grown for 24 h before the experiments. The siRNA assays were carried out using the PKCδ siRNAs, control siRNA and transfection reactives from Santa Cruz Biotechnology. For siRNA transfection, the cells were seeded out without antibiotics, grown for 24 h and transfected according to the manufacturer's procedure. After 5 h of transfection, the medium was changed to complete growth medium containing serum and antibiotics, and the cells were grown for 2 or 3 days. Inactivation of PKCδ was also accomplished by incubating HeLa cells in the presence of 25 μM rottlerin (Calbiochem) for 60 min. After silencing or inactivating PKCδ, DNA damage was induced by incubating the cells in the presence of 0.02% MMS for 1 h.

### Western blot analysis

Yeast total protein extracts were prepared as previously described (39). For HeLa total protein extracts, cells were collected in 100 μl of lysis buffer (20 mM Tris-HCl pH 8.0, 100 mM NaCl, 0.5% Triton X-100, 10 mM NaF, 0.1 mM Na_3_V_4_, 1 M phenylmethanesulfonylfluoride (PMSF)) supplemented with a mixture of protease inhibitors (Roche) and the lysates were sonicated for 10 s. After centrifugation, 100 μl of sample buffer (0.2 M Tris-HCl pH 7.5, 8% sodium dodecyl sulphate (SDS), 40% glycerol, 0.2 M dithiothreitol (DTT), 0.04% bromophenol blue) were added to the cleared supernatants and samples were incubated for 5 min at 95°C. Equivalent amounts of protein were resolved in an SDS-polyacrylamide gel electrophoresis gel and transferred onto a nitrocellulose membrane. The primary antibodies used in this study include anti-Rad53–YC19 (Santa Cruz Biotechnology), anti-GFP (Roche), anti-Histone H2A (phospho S129) (Abcam), anti-Histone H2A (Active Motif), anti-HA 3F10 monoclonal antibody (Roche), anti-yeast PKC1 (yC-20) (Santa Cruz Biotechnology), anti-PKCδ C-20 (Santa Cruz Biotechnology), anti-phospho-Chk2 (Thr68), anti-Chk2 (Santa Cruz Biotechnology) anti-β-actin (Santa Cruz Biotechnology). Blots were developed with horseradish peroxidase-labeled secondary antibodies using the ECL Advance Western Blotting Detection Kit (GE Healthcare Life Sciences) and an ImageQuant^TM^ LAS 4000mini Biomolecular Imager (GE Healthcare).

### Dephosphorylation assay

Yeast total protein extracts were prepared by vigorous shaking of cells in the presence of glass beads in a non-denaturing lysis buffer (150 mM KCl, 50 mM Tris-HCl pH 8.0, 20% glycerol, protease inhibitors cocktail from MERCK Millipore). The dephosphorylation assays were carried out by incubating 10 mg of cell extract in λ protein phosphatase buffer (50 mM Tris-HCl pH 7.8, 5 mM DTT, 2 mM MnCl2, 100 μg/ml bovine serum albumin) containing 200 U of λ protein phosphatase (MERCK Millipore) for 20 min at 30°C. Phosphatase reactions were stopped by adding an equal volume of 2x western sample buffer and incubating at 95°C for 2 min.

### DNA content analysis

Approximately 10^6^ cells were fixed in 50% EtOH. Fixed cells were treated overnight at 30º with 0.1 mg/ml ribonuclease A in 50-mM sodium citrate buffer. Finally, cells were stained with 20 μg/ml propidium iodide. DNA content was analyzed in a BD FACSVerse (Becton, Dickinson and Company) cytometer.

### Fluorescence microscopy

Pkc1-GFP protein was visualized in living cells grown in synthetic media and analyzed in an *Axioskop 2 Zwiss inc* microscopy. Images were captured with an *AxioCam MRm* (Zeiss Inc.) camera and *Axio Vision v4.7* (Zeiss Inc.).

## RESULTS

### Pkc1 is required for DNA integrity checkpoint activation

**Figure 1.**
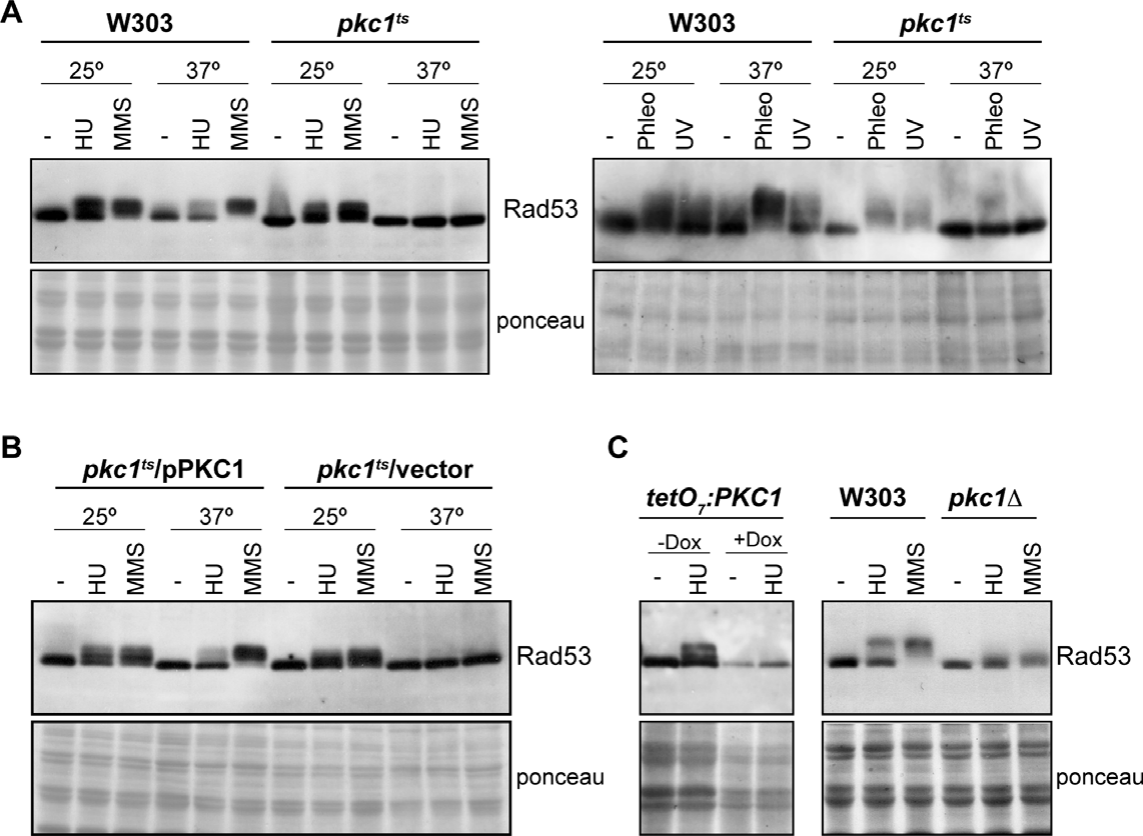
Analysis of Rad53 checkpoint kinase activation in *pkc1* mutant strains. Activation of the checkpoint kinase Rad53 was determined by western analysis as slower migrating bands using an anti-Rad53 antibody. The ponceau staining of the membrane is shown as loading control. (**A**) Exponentially growing cultures of the wild-type (W303-1a) and *pkc1^ts^* (JC6-3a) strains were split and incubated for three hours at 25º or 37º followed by 1 h incubation in the absence or presence of 0.2 M HU, 0.04% MMS or 5 μg/ml phleomycin, or were irradiated with 50 J/m^2^ UV radiation. (**B**) Exponentially growing cultures of the *pkc1^ts^* (JC6-3a) strain transformed with a centromeric plasmid containing the *PKC1* gene or an empty vector were assayed as described in (A). (**C**) Exponentially growing cultures of the *tetO_7_:PKC1* (JCY1471) strain incubated for 8 h in the absence or presence of 5 μg/ml doxycycline and the wild-type (W303-1a) and *pkc1Δ* (GPY1115) strains grown in the presence of 1 M sorbitol were incubated for 1 h in the absence or presence of 0.2 M HU or 0.04% MMS as indicated.

Genomic stability depends on proper DNA integrity checkpoint function. Therefore, in a first attempt to clarify the connection between Pkc1 and genomic integrity, we investigated whether the checkpoint functionality was related to Pkc1 activity by analyzing the activation of checkpoint kinase Rad53 in *pkc1* mutant cells. Activation of Rad53 was tested by western analysis since Rad53 phosphorylation in response to genotoxic treatments results in the appearance of slower migration forms of the protein. When Rad53 activation was analyzed in a thermosensitive *pkc1* mutant (*pkc1^ts^*) after treating cells with various genotoxic agents, such as HU, MMS, phleomycin or UV radiation, phosphorylated forms of Rad53 were detected at 25ºC, similar to that observed in the wild-type strain. However, no activation of Rad53 was observed at 37ºC (Figure 1A). It is important to note that the activation of Rad53 was restored in the *pkc1* mutant cells transformed with a centromeric plasmid containing the *PKC1* gene (Figure 1B), which confirms that Pkc1 activity is required for checkpoint kinase Rad53 activation in response to DNA damage. It should also be noted that *pkc1-8* mutation is not lethal at 37ºC (48). Although growth is compromised compared to wild type, *pkc1-8* cells proliferate and are not arrested at a cell cycle stage (Supplementary Figures S1 and S4). Moreover, defects in Rad53 activation were also observed in other *pkc1* mutant strains, such as the strains containing either the *tetO_7_:PKC1* gene, whose expression turns off in the presence of doxycycline, or the *pkc1Δ* allele (Figure 1C). In summary, the analysis of Rad53 activation in various *pkc1* mutant strains revealed a direct connection between PKC and the DNA integrity checkpoint, where Pkc1 is necessary for proper checkpoint activation.

**Figure 2.**
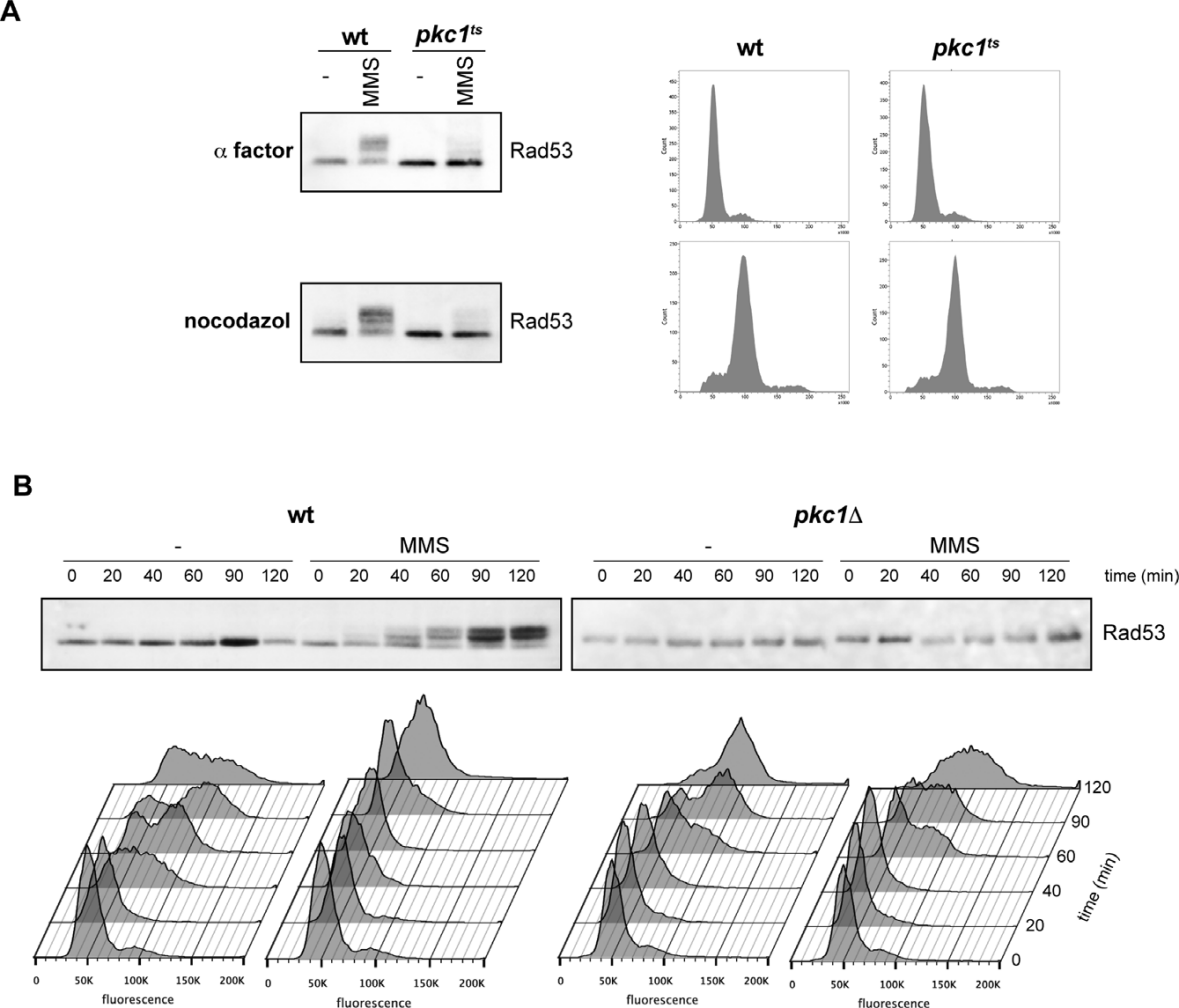
Analysis of DNA damage checkpoint function at different cell cycle stages. (**A**) α-factor or nocodazole was added to exponentially growing cultures of the wild-type (W303-1a) and *pkc1^ts^* (JC6-3a) strains in order to block cell cycle progression at the G1 or G2/M phase, respectively. After 30 min, cells were transferred to 37º and incubated for 3 h followed by 1 h incubation in the absence or presence of 5 μg/ml phleomycin. Activation of the checkpoint kinase Rad53 was determined by western analysis. Cell cycle arrest was confirmed by cell morphology (more than 95% of unbudded cells or more than 90% of large budded cells, respectively) and analysis of DNA content by flow cytometry (right panels). (**B**) α-factor arrested cells of the wild-type (W303-1a) and *pkc1Δ* (GPY1115) strains were released into YPDsorbitol medium in the absence or presence of 0.02% MMS. At the indicated times, activation of the checkpoint kinase Rad53 and cell cycle progression were investigated by western blot or analysis of DNA content by flow cytometry, respectively.

Cells respond to DNA damage at different stages of cell cycle. Therefore, to complete the study of checkpoint functionality in *pkc1* mutant cells, we investigated checkpoint activation when DNA damage was inflicted in G1, S or G2/M phase of the cell cycle. First, cells were arrested in G1 or G2/M by incubation in the presence of α-factor or nocodazole, respectively, and DNA damage was induced by the addition of phleomycin, a treatment that it is known to activate checkpoint outside the S phase. As it can be observed in Figure 2A, inactivation of Pkc1 severely impaired the activation of DNA damage checkpoint in G1 and G2/M. On the other hand, α-factor-blocked cells were released from the arrest in the presence of MMS in order to induce a checkpoint response during S phase. Whereas wild-type cells activated Rad53 kinase and halted cell cycle progression, *pkc1* mutant cells failed to properly activate the checkpoint and to block cell cycle progression (Figure 2B). All these observations are consistent with the results described above in asynchronous cultures and reveal that Pkc1 activity is involved in the activation of DNA integrity checkpoint in response to damage in all cell cycle stages.

Pkc1 controls the Slt2 MAPK kinase cascade. Previous results from our group have demonstrated that MAPK Slt2 is not required for checkpoint activation (39). We completed this study by investigating whether other kinases of the cascade are involved in Rad53 activation. Similar to what was observed for Slt2, Rad53 phosphorylation occurs in the mutant strains in MAPKKs Mkk1, 2 or MAPKKK Bck1 (Supplementary Figure S2). Thus, the function in checkpoint activation is a specific role played by Pkc1 that is independent of the MAPK cascade.

### DNA integrity checkpoint control by Pkc1 involves its kinase activity

**Figure 3.**
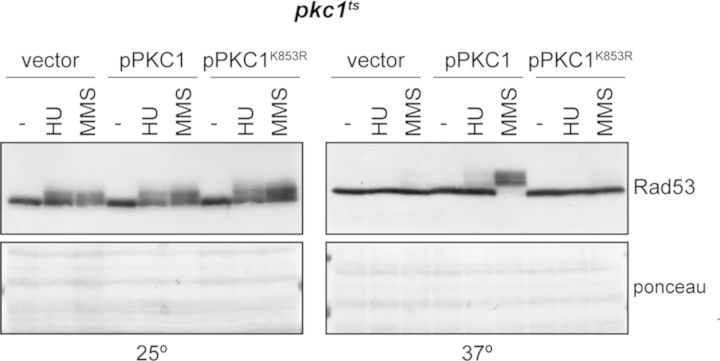
Analysis of Rad53 checkpoint kinase activation by a kinase-dead Pkc1 mutant protein. Exponentially growing cultures of the *pkc1^ts^* (JC6-3a) strain transformed with a centromeric plasmid containing the *PKC1*, the *PKC1^K853R^* (kinase-dead mutation) gene or an empty vector were split and incubated for 3 h at 25º or 37º followed by 1 h incubation in the absence or presence of 0.2 M HU or 0.04% MMS. Activation of the checkpoint kinase Rad53 was determined by western analysis. The ponceau staining of the membrane is shown as loading control.

Next, we tested whether Pkc1 kinase activity is required for proper checkpoint activation in response to DNA damage. Rad53 activation was checked in *pkc1* mutant cells expressing a kinase-dead mutant protein, Pkc1^K853R^ (49). Whereas the expression of the wild-type *PKC1* gene was able to sustain Rad53 activation, no Rad53 phosphorylation was observed in the cells expressing the Pkc1^K853R^ mutant protein (Figure 3). This indicates that the kinase activity of Pkc1 is required for checkpoint control in response to DNA damage, suggesting that Pkc1 may act by phosphorylating essential targets for Rad53 activation.

### Pkc1 activity mediates the activation of Mec1 and Tel1 in response to genotoxic stress

The above results indicate that there is not activation of checkpoint kinase Rad53 after genotoxic stress if the Pkc1 function is absent. In *S. cerevisiae*, Rad53 phosphorylation in response to different types of damage depends mainly on the apical checkpoint kinase Mec1, whereas Tel1 plays a more secondary role. To test if the defects observed in Rad53 activation in the *pkc1* mutant strains are specific of Rad53 or if they reflect defects in Mec1 or both Mec1 and Tel1 kinases, DNA damage was induced and other phosphorylation events that are dependent on Tel1 kinase, such as the phosphorylation of histone H2A and the MRX complex protein Xrs2, were analyzed.

**Figure 4.**
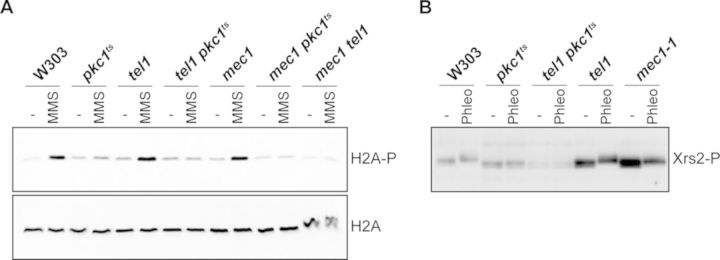
Analysis of Mec1- and Tel1-dependent H2A and Xrs2 phosphorylation in *pkc1* mutant strains. (**A**) Exponentially growing cultures of the wild-type (W303-1a), *pkc1^ts^* (JC6-3a), *tel1* (JCY1258), *tel1 pkc1^ts^* (JCY1271), *mec1* (JCY1039), *mec1 pkc1^ts^* (JCY1352) and *mec1 tel1* (JCY1275) strains were incubated for 3 h at 37º followed by 1 h incubation in the absence or presence of 0.06% MMS. The level of phosphorylated H2A and total H2A protein was determined by western analysis using an antibody specific for H2A phosphorylated in Ser129 or an anti-H2A antibody, respectively. (**B**) Exponentially growing cultures of the wild-type (W303-1a), *pkc1^ts^* (JC6-3a), *tel1* (JCY1258), *tel1 pkc1^ts^* (JCY1271) and *mec1-1* (N1-3a) strains transformed with the pXRS2-HA plasmid were incubated for 3 h at 37º followed by 1 h incubation in the absence or presence of 25 μg/ml phleomycin. Phosphorylation of Xrs2 was determined by western analysis as slower migrating bands.

For the histone H2A, it has been described that genotoxic stress induces the phosphorylation of Ser129 over a 50–100-kb region around the damage site and that this phosphorylation can be carried out by both Mec1 and Tel1 kinases (50–52). In order to examine whether the defects observed in checkpoint activation in the absence of Pkc1 were due to the defective activation of Mec1 and/or Tel1, we studied if H2A was properly phosphorylated in response to genotoxic stress in the *pkc1* mutant cells. The results show that Ser129 phosphorylation in response to DNA damage can still be detected in single *mec1* or *tel1* mutant cells, and it is abolished only in *mec1 tel1* double mutant strain (Figure 4A). Interestingly, H2A phosphorylation was drastically reduced in the absence of Pkc1 activity. The level of H2A phosphorylation observed in the *pkc1* mutant strains was weaker than that detected in single *mec1* and *tel1* mutants, suggesting that both kinases are affected by Pkc1 inactivation. However, it has to be noted that the level of phosphorylated H2A detected in *pkc1* mutant cells was somewhat higher than that observed in the double *mec1 tel1* mutant strain. This may be due to the presence of residual Mec1 and/or Tel1 activity, which might be able to drive a basal level of H2A phosphorylation. In summary, cells need fully functional Pkc1 to correctly drive Mec1- and Tel1-dependent phosphorylation of histone H2A in response to DNA damage.

The Xrs2 protein constitutes with Mre11 and Rad50 the MRX complex, which plays an important role in the processing of DSBs. Xrs2 is phosphorylated during the cellular response to DNA damage preferentially in a Tel1-dependent manner (15,53,54). We studied whether the absence of Pkc1 activity affects Xrs2 phosphorylation in asynchronous cells treated with phleomycin. In our experimental conditions, Xrs2 phosphorylation (detected by western blot as retarded migration forms) was not significantly altered in either the *mec1* or the *tel1* single mutant strains (Figure 4B). In contrast, when the functional Pkc1 was absent, as in the *pkc1* and *pkc1 tel1* cells, no phosphorylated forms of Xrs2 were detected, similar to what happened in *mec1 tel1* double mutant strains (53,54). Therefore, Pkc1 activity is needed to allow the Mec1- and Tel1-dependent phosphorylation of Xrs2 in response to phleomycin.

Overall, the fact that Rad53, H2A and Xrs2 phosphorylation is severely affected after DNA damage when a functional Pkc1 is absent allows us to conclude that Pkc1 activity is necessary for the correct activation of both the Mec1 and Tel1 checkpoint kinases in response to DNA damage.

### Pkc1 delocalizes in response to genotoxic stress

**Figure 5.**
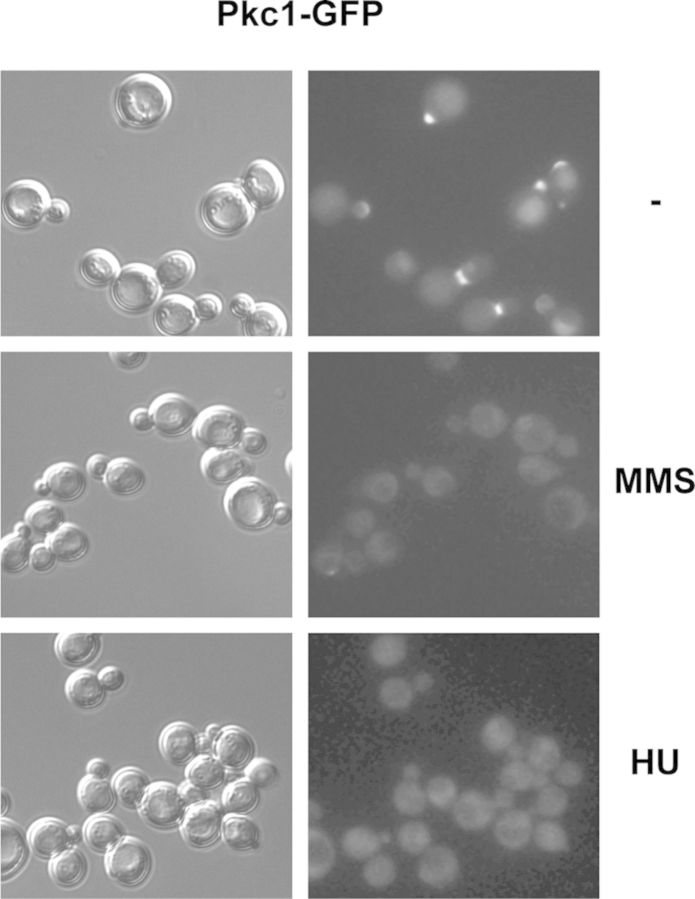
Analysis of Pkc1 subcellular localization after genotoxic stress. Exponentially growing wild-type cells expressing a GFP-tagged Pkc1 protein (JCY1511) were incubated for 1 h in the absence or presence of 0.2 M HU or 0.04% MMS. Differential interference contrast (DIC) image and GFP fluorescence signal are shown.

The best known function of Pkc1 is cell wall integrity maintenance in response to polarized cell growth and to various environmental stresses. Not surprisingly, Pkc1 localizes at sites of polarized growth, specifically at the bud tip in early cell cycle stages, and at the mother-bud neck during cytokinesis (55). The function of Pkc1 in the DNA integrity checkpoint suggests that a change in Pkc1 localization may occur after genomic damage. Consequently, localization of GFP-tagged Pkc1 was investigated in response to HU or MMS treatments. The results show that, as expected, Pkc1 was located in the bud neck and bud tip of cells in the absence of drugs (Figure 5). However, Pkc1 disappeared from the sites of polarized growth when cells were treated with HU or MMS. It is important to remark that the change in localization was observed as early as 30 min, an incubation time at which polarized growth still occurred and dumbbell cells had not yet accumulated. This suggests that relocation of Pkc1 in response to replication stress might be a direct response to HU or MMS and may not be caused by lack of polarized growth. The delocalization of Pkc1 also occurred in the *mec1* and *tel1* mutant strains, suggesting that Pkc1 relocation is not governed by the checkpoint signaling (Supplementary Figure S3).

It is plausible to expect that the role played in checkpoint activation is a nuclear function of Pkc1. However, no nuclear accumulation of Pkc1 was observed under the genotoxic stress conditions, although it cannot be ruled out that a small fraction of the protein could be translocated inside the nucleus. Several checkpoint proteins associate with the site of damage. We investigated this possibility for Pkc1 after the induction of a single DSB at a specific site by means of the *GAL1:HO* strain, which expresses the HO endonuclease after adding galactose to the growth medium (56). Unlike what occurred for the Ddc2 protein (57), we were unable to detect nuclear Pkc1 foci by fluorescence microscopy or binding to DNA by chromatin immunoprecipitation after the induction of a single DSB (Supplementary Figure S4).

### Pkc1 is post-translationally modified in response to DNA damage in a Tel1-dependent way

**Figure 6.**
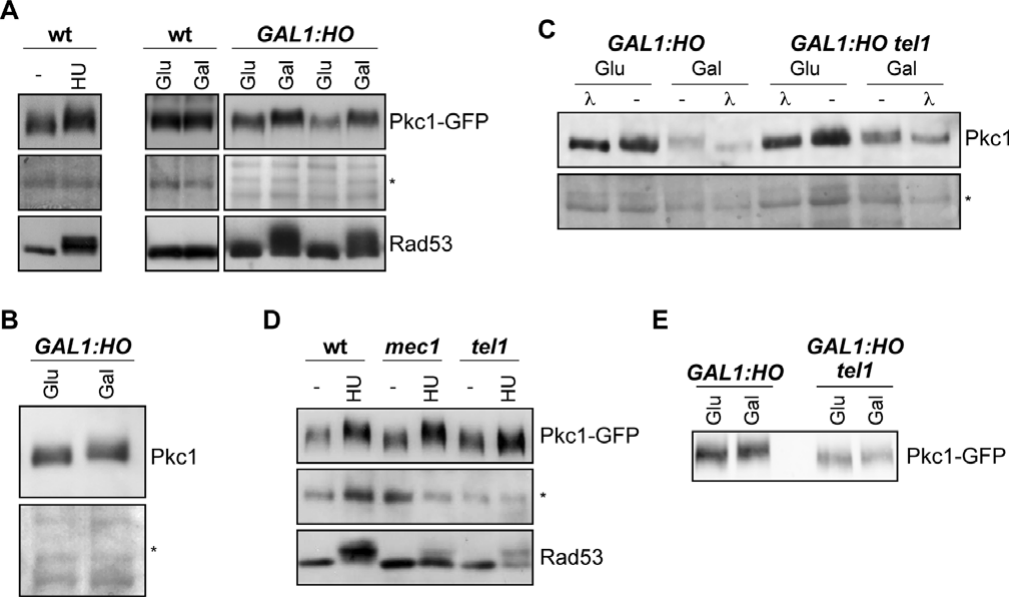
Analysis of Pkc1 electrophoretic mobility after induction of DNA damage. (**A**) Left panel: exponentially growing wild-type cells expressing a GFP-tagged Pkc1 protein (JCY1511) were incubated for 2 h in the absence or presence of 0.2 M HU; right panel: raffinose-grown wild-type (JCY1511) and *GAL1:HO* (JCY1514) cells expressing a GFP-tagged Pkc1 protein were incubated for 4 h in the presence of 2% glucose or 2% galactose. Electrophoretic mobility of Pkc1-GFP was investigated by western analysis using an anti-GFP antibody. An unspecific band in the western blot or the ponceau staining of the membrane is showed as a control of migration. Activation of the checkpoint kinase Rad53 is showed as a control of DNA damage induction. (**B**) Raffinose-grown *GAL1:HO* (JKM139) cells were incubated for 4 h in the presence of 2% glucose or 2% galactose. Electrophoretic migration of endogenous Pkc1 was investigated by western analysis using an anti-yeast Pkc1 antibody. (**C**) Raffinose-grown *GAL1:HO* (JKM139) and *GAL1:HO tel1* (JCY1654) cells were incubated for 4 h in the presence of 2% glucose or 2% galactose. Electrophoretic migration of endogenous Pkc1 after incubation of cell extracts in the absence or presence of λ protein phosphatase was analyzed as in (B). (**D**) Electrophoretic migration of Pkc1-GFP in wild-type (W303-1a) *mec1* (JCY1580) and *tel1* (JCY1583) cells after addition of HU was analyzed as described in (A). (**E**) Raffinose-grown *GAL1:HO* (JKM139) and *GAL1:HO tel1* (JCY1654) cells transformed with pPKC1-GFP plasmid were incubated for 4 h in the presence of 2% glucose or 2% galactose. Electrophoretic migration of Pkc1-GFP was investigated by western analysis using an anti-GFP antibody.

Genotoxic damage leads to the phosphorylation of Slt2 MAPK of the PKC pathway (39). We wondered whether Pkc1 could also be modified in response to genotoxic stress. To analyze this, the electrophoretic mobility of Pkc1 was analyzed in extracts from cells treated with HU or after the induction of a single DSB by means of the *GAL1:HO* strain. It is interesting to note that both treatments caused a delay in the electrophoretic mobility of Pkc1 (Figure 6A). No change in mobility was detected after the shift to galactose for the control strain lacking the *GAL1:HO* gene, indicating that the band shift is a genuine response to DNA damage (Figure 6A). Importantly, the band shift was observed with both Pkc1-GFP and the endogenous Pkc1 protein (Figure 6B). These results indicate that Pkc1 undergoes some kind of post-translational modification in response to genotoxic treatments.

In order to determine the nature of the post-translational modification of Pkc1, we carried out a dephosphorylation assay with cell extracts from the *GAL1:HO* strain. It is known that Pkc1 is a phosphoprotein (49,58,59). Consistent with this, incubation of proteins from control cells with λ protein phosphatase resulted in a faster migration form of Pkc1 (Figure 6C). Interestingly, treatment of cell extracts from cells after induction of DSB resulted in the disappearance of slower migration forms and the presence of a Pkc1 form with the same electrophoretic mobility as from control cells. This result indicates that the post-translational modification suffered in response to induction of DSB is a phosphorylation event.

The cellular response to genotoxic stress is governed by the DNA integrity checkpoint. To address whether the checkpoint kinases were involved in Pkc1 phosphorylation, the electrophoretic mobility of Pkc1 was studied in mutant cells in either Mec1 or Tel1 kinases. As observed in Figure 6D, genotoxic treatments still induced a change in Pkc1 mobility when Mec1 was absent. Yet when Tel1 was inactivated, no change in electrophoretic mobility was observed (Figure 6C–E). Thus, Tel1, but not Mec1, mediates phosphorylation of Pkc1 in response to genotoxic stress.

### Expression of mammalian PKCδ in *pkc1* mutant cells suppresses the defect in Rad53 activation by DNA damage

Key cellular mechanisms are conserved in eukaryotes from yeast to mammals. This is particularly remarkable in cell cycle and checkpoint control. We wondered whether mammalian PKCs could also control the DNA integrity checkpoint. Accordingly, we expressed in yeast *pkc1* mutant cells different isoforms of mouse PKCs representing distinct PKC subfamilies: conventional PKCα, novel PKCδ, PKCε and PKCη, atypical PKCι and PKC-related kinase PRK2. Mammalian PKCs were expressed under the control of the *PKC1* promoter in order to mimic the expression of endogenous Pkc1. No isoform tested was able to support growth of the *pkc1^ts^* cells at the restrictive temperature of 38ºC (Supplementary Figure S5). Cell death in the absence of Pkc1 is due to cell lysis caused by defects in cell wall biosynthesis (60,61). Therefore, our result indicates that mammalian PKCs are unable to support the essential function in the maintenance of yeast cell integrity, at least when expressed at endogenous levels.

**Figure 7.**
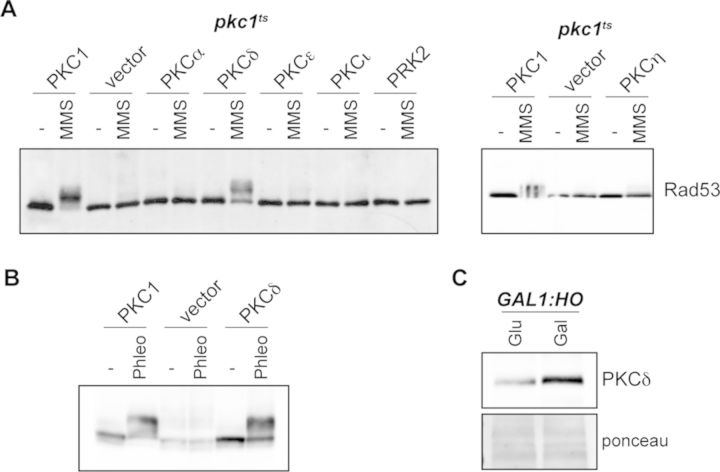
Analysis of the activation of the yeast Rad53 checkpoint kinase by mammalian PKC isoforms. (**A**) Exponentially growing cultures of the *pkc1^ts^* (JC6-3a) strain transformed with an empty vector or a centromeric plasmid expressing yeast *PKC1* or mammalian PKCα, PKCδ, PKCε, PKCι, PKCη or PRK2 (under the control of *PKC1* promoter) were incubated for 3 h at 37º followed by 1 h incubation in the absence or presence of 0.04% MMS. Activation of the checkpoint kinase Rad53 was determined by western analysis. (**B**) Exponentially growing cultures of the *pkc1^ts^* (JC6-3a) strain transformed with an empty vector or a centromeric plasmid expressing yeast *PKC1* or mammalian PKCδ were incubated for 3 h at 37º followed by 1 h incubation in the absence or presence of 5 μg/ml phleomycin. (**C**) Raffinose-grown *GAL1:HO* (JCY1514) cells transformed with the pPKCδ plasmid were incubated for 4 h in the presence of 2% glucose or 2% galactose. Electrophoretic mobility of PKCδ was investigated by western analysis using an anti-PKCδ antibody. The ponceau staining of the membrane is showed as a control of migration.

Next, we checked Rad53 activation by MMS in the yeast cells expressing mammalian PKCs. Interestingly one of them, PKCδ, was able to support checkpoint activation in yeast cells (Figure 7A). We also tested another genotoxic agent such as phleomycin (Figure 7B). In all cases, no differences were noted in the checkpoint activation driven by yeast Pkc1 or mammalian PKCδ. Furthermore, PKCδ also underwent an electrophoretic mobility band shift when a DSB was induced (Figure 6C), similar to what was observed for Pkc1. PKCδ is 34.0% identical to Pkc1 (48.4% identical at the C-terminal catalytic domain and 21.6% identical at the N-terminal regulatory domain). In conclusion, our results demonstrate that mammalian PKCδ performs a biological function related to the maintenance of the genome integrity in response to DNA damage and that this function is conserved with the yeast Pkc1 protein.

### PKCδ is required for proper DNA damage checkpoint activation in human cells

**Figure 8.**
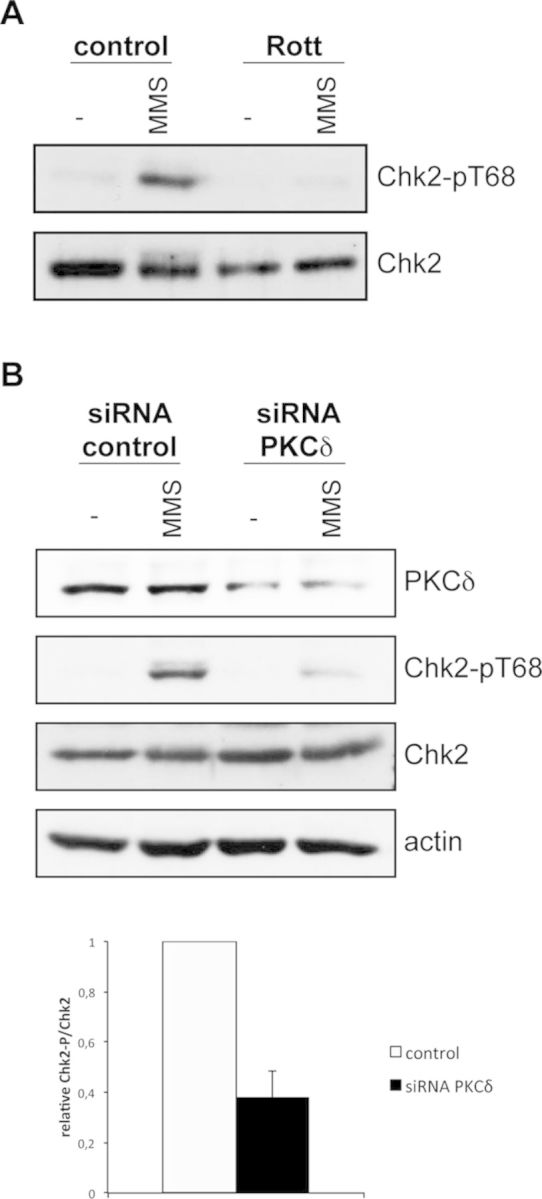
Analysis of the activation of the mammalian Chk2 kinase by PKCδ in response to DNA damage. (**A**) HeLa cells were incubated for 45 min in the absence or presence of 25 μM rottlerin followed by 1 h incubation in the presence of 0.02% MMS. Protein level of total and Thr68-phosphorylated Chk2 was analyzed by western blot. (**B**) Hela cell cultures were split and transfected with a siRNAPKCδ or a siRNAcontrol. After transfection, cells were incubated in the presence of 0.02% MMS for 1 h. Protein level of PKCδ, total and Thr68-phosphorylated Chk2 and actin was determined by western analysis. The graph represents mean and standard deviation of the relative ratio of phosphorylated Chk2 protein derived from four independent assays.

The Chk2 protein is the human ortholog of Rad53. It is known that Chk2 is activated in the presence of genomic damage by the phosphorylation of threonine 68 by ATM. Given PKCδ's ability to recover DNA damage-induced Rad53 activation in yeast *pkc1* mutant cells, we decided to analyze whether DNA damage-induced Chk2 activation in human cells also depends on PKCδ. In a first approach, Chk2 phosphorylation in Thr68 was analyzed after treating HeLa cells with MMS in the absence or presence of rottlerin, a compound often used as an inhibitor of the PKCδ isoform. The results shown in Figure 8A indicate that Chk2 activation after DNA damage was impeded when cells were incubated with rottlerin. This result suggests that, as in yeast, mammalian PKC, particularly PKCδ, may be needed to activate the DNA damage checkpoint.

Despite the fact that rottlerin has been widely used as a specific inhibitor of PKCδ in many works, it has been reported that it may also inhibit other cellular kinases depending on the dose used (62,63). Therefore, in order to clarify whether PKCδ is indeed involved in DNA damage checkpoint activation, the specific inhibition of PKCδ was achieved by ribonucleic acid (RNA) interference. Thus, HeLa cells were transfected with a control siRNA or PKCδ siRNA and were incubated in the presence or absence of MMS. Western analysis showed a significant reduction in the PKCδ protein levels in those cells containing the PKCδ siRNA when compared to those treated with the control siRNA, which confirmed that interference had indeed taken place, be it partially. Importantly, the achieved PKCδ silencing sufficed to give rise to an ∼60% decrease in the level of MMS-induced Chk2 activation (Figure 8B). This observation allowed us to conclude that, as in yeast cells, PKCδ is required for proper DNA damage checkpoint activation in human cells.

## DISCUSSION

Previous published results have suggested a possible connection of Pkc1 kinase with DNA metabolism. Our group described a genetic interaction of *PKC1* with mutations in replication cyclin Clb5 and checkpoint adaptor protein Rad9 (38). Furthermore, the *pkc1* mutant exhibits a high recombination rate, which is a typical trait of defects in genomic integrity (41). Moreover, *pkc1* mutant strain is hypersensitive to different genotoxic agents such as HU, bleomycin, MMS and 4NQO (38,42,43). In this work, we have established a clear direct connection of PKC with DNA metabolism: Pkc1 activity is required in yeast cells to activate the DNA integrity checkpoint. We have checked this effect in different independent backgrounds and, moreover, checkpoint activation was restored when a *PKC1* gene was re-introduced into *pkc1* mutant cells, demonstrating that the lack of checkpoint function is caused by Pkc1 inactivation. The fact that a Pkc1 mutant protein lacking its kinase activity was unable to restore the checkpoint function strongly suggests that Pkc1 must act by phosphorylating key targets involved in checkpoint activation. Furthermore, failure of the DNA integrity checkpoint in *pkc1* mutant cells is observed in response to a wide range of genotoxic stresses, such as double-stranded chromosomal breakage, thymine dimers, nucleotide alkylation or replication forks stalling, indicating that Pkc1 plays a central role in the cellular response to genomic damage.

Pkc1 controls the cell integrity regulating the Slt2 MAPK kinase cascade. It is important to remark that while Pkc1 activity is required for the activation of DNA integrity checkpoints, this is not the case for the MAPK cascade since Rad53 phosphorylation is detected in mutant strains in all the kinases of the cascade. Consistently with our results, it was described that the *pkc1* mutant, but not the *slt2* mutant, displays a high mitotic recombination rate (41). This phenotypic trait is characteristic of mutants that perform important functions in DNA metabolism since defects in these proteins lead to increased DNA damage and recombination events. All these observations indicate that Pkc1 regulates DNA integrity by a pathway other than the MAPK module and the mechanism used in cell integrity control. Consistent with this idea, growth defect (60,61) but not the hyper-recombination phenotype (41) nor the impaired DNA integrity checkpoint function (this work) can be reversed by osmostabilizing agents. It has to be noted that *pkc1Δ* cells are viable when grown on sorbitol in spite of the defective checkpoint. Mec1 and Rad53 are essential proteins; therefore, the viability of *pkc1Δ* cells could be due to the existence of a residual checkpoint activity in the absence of Pkc1 supported by other cellular pathways acting on sorbitol grown cells or, alternatively, that checkpoint proteins might carry out other functions independently of Pkc1. Supporting this, we have observed that checkpoint mutant cells are more sensitive to genotoxic treatments than *pkc1* cells (Supplementary Figure S6).

At what level does Pkc1 control the DNA integrity checkpoint? We have observed severe defects in the phosphorylation of checkpoint kinase Rad53, histone H2A and MRX subunit Xrs2. These phosphorylation events depend on a functional checkpoint and, at least for the two last proteins, their phosphorylation in response to damage is severely affected only in double mutant strains in both Mec1 and Tel1 kinases. Hence, our results indicate that Pkc1 activity is necessary for both Mec1- and Tel1-dependent phosphorylation events in response to genotoxic stresses. It cannot be ruled out that Pkc1 may act on checkpoint substrates by a parallel branch independently of Mec1/Tel1. Nonetheless, the fact that multiple substrates are affected enabled us to propose a model in which Pkc1 would act upstream of Mec1/Tel1 to directly or indirectly control their activation (Supplementary Figure S7).

One observation that revealed another aspect of the connection between Pkc1 and the cellular response to DNA damage was the electrophoretic band shift observed for Pkc1 in cells under genotoxic stress, particularly a DSB and replicative stress. This protein migration change reflects the phosphorylation of Pkc1 and strongly suggests that Pkc1 activity is regulated by genotoxic stress. This hypothesis is supported by a report indicating that HU activates PKC in mammalian cells (64) and that PKCδ is phosphorylated by c-Abl and ATM kinases in response to DNA damage (65,66). Checkpoint kinases are putative candidates to modify Pkc1 protein. We determined that Pkc1 post-translational modification depends specifically on Tel1, but not on Mec1. This result allowed us to complete the proposed model to the extent that in the presence of DNA damage, there would be a feedback loop in which Tel1, whose activation requires Pkc1, would be responsible for regulating the Pkc1 protein. Tel1 mediates the response to DSB, so this feedback mechanism might play a relevant role in the specific response to DSB. Further work will be required to elucidate the exact nature and function of this Tel1-Pkc1 feedback mechanism.

Checkpoint activation occurs *in situ* at the site of DNA damage. Since Pkc1 controls such activation, it seemed logical to believe that Pkc1 should move into the nucleus in response to genotoxic treatments in order to access their targets. It is important to remark that four putative nuclear localization signals (NLS) have been described in Pkc1 and that nuclear import activity has been demonstrated for at least one of them (67). These observations suggest that Pkc1 must play a role inside the nucleus. Pkc1 has been localized at sites of polarized growth, which reflects its major function in the maintenance of cell integrity. We observed the mobilization of Pkc1 outside these sites after genotoxic stress. However, we were unable to detect whether this change reflects a relocalization of the protein inside the nucleus and its association with the site of damage. It cannot be ruled out that a fraction of the protein below the technique's limit of detection indeed enters the nucleus to activate checkpoint kinases. Some proteins that are known to bind to damaged DNA, such as Rad24, Rad9 and Rad53, have been barely detected at the DSB site using the same chromatin immunoprecipitation approach described here, suggesting a transient or substoichiometric binding to the lesion site (57). This could also be the case for Pkc1. Alternatively, Pkc1 could carry out an essential step required for Mec1 and Tel1 activation in the cytoplasm.

Mammalian cells contain multiple PKC isoforms that differ in terms of their function and regulation. As the proteins that perform important cellular functions are evolutionarily conserved from yeast to humans, we wondered whether some mammalian PKCs are functionally related to yeast Pkc1. In *S. cerevisiae*, the absence of Pkc1 causes death due to cell lysis and, as described herein, defects in DNA integrity checkpoint activation. It has been previously reported that PKCη can partially suppress the growth defect of *pkc1* mutant cells (68). However, we were unable to suppress the lethality of the *pkc1* mutation with either PKCη or any other mammalian isoforms tested. This discrepancy could be due to the distinct *pkc1* mutant strains used, or is more likely due to differences in the expression level of PKCη: while we expressed it from the *PKC1* promoter to guarantee a similar protein level to that of endogenous Pkc1, Nomoto *et al.* (68) highly overexpressed PKCη from the *GAL1* promoter. On the other hand, PKCδ was the only mammalian isoform able to restore checkpoint activation by genotoxic damage when expressed in yeast *pkc1* mutant cells. These results demonstrate that PKC performs cellular functions in morphogenetic processes and in the DNA integrity maintenance that are conserved from yeasts to humans. These functions have been specialized between specific isoforms in mammalian cells: PKCη may be related to the Pkc1 function in cell integrity maintenance whereas PKCδ is related to the Pkc1 function in DNA integrity maintenance.

One particularly interesting aspect of this work is the case of PKCδ. First, the fact that PKCδ perfectly regulates Rad53 in yeast cells reveals that the molecular mechanism underlying DNA integrity checkpoint control by Pkc1 is conserved from yeast to mammals. Supporting this, the Tel1-dependent band shift observed for Pkc1 in response to DSB was also observed for PKCδ when expressed in yeast. Besides, PKCδ was phosphorylated by ATM, the mammalian ortholog of Tel1 (65). More importantly, we demonstrate that PKCδ activity is relevant for Chk2 (mammalian ortholog of Rad53) activation by DNA damage in human cells. This observation reveals a new crucial role of PKCδ in correct DNA damage checkpoint functioning in human cells. To date it is known that PKCδ takes part in the induction of apoptosis in response to DNA damage (69). In the presence of DNA damage, the ATM checkpoint kinase functions upstream of PKCδ activation. As a result, PKCδ translocates into the nucleus where it phosphorylates the hRad9 protein, a component of the 9-1-1 checkpoint clamp complex. This favors Rad9 interaction with anti-apoptotic protein Bcl-2, thereby activating apoptosis (65). In addition, PKCδ induces apoptosis in response to DNA damage through the regulation of p53 protein and by other p53-independent mechanisms (70,71). Our results reflect a more complex relation between PKCδ and the DNA damage response since PKCδ is also required to properly activate the Chk2 kinase. Pkc1 controls Mec1 and Tel1 activity in yeast, and PKCδ is able to perform the same function in yeast. This leads us to believe that the defect observed in DNA damage-induced Chk2 phosphorylation when PKCδ activity is inhibited reflects a defect in ATM and ATR kinases. In short, our observations indicate that DNA integrity checkpoint control by PKC is not restricted to yeast, but must be a general trait of eukaryotic cells. Studies into simpler organisms like yeast or in humanized yeast expressing PKCδ can definitively help our understanding of the cellular functions performed by PKC in DNA integrity checkpoint control in more complex systems like mammals.

## SUPPLEMENTARY DATA

Supplementary Data are available at NAR Online.

SUPPLEMENTARY DATA
