# Supplementary material for: Protein kinase C controls activation of the DNA integrity checkpoint
Source: Nucleic Acids Res. 2014 May 3;42(11):7084–95. doi: 10.1093/nar/gku373 (PMC4066786; doi:10.1093/nar/gku373)
Supplement: SUPPLEMENTARY DATA [file supp_42_11_7084__index.html]

Protein kinase C controls activation of the DNA integrity checkpoint — Protein kinase C controls activation of the DNA integrity checkpoint — SUPPLEMENTARY DATA 

# Protein kinase C controls activation of the DNA integrity checkpoint

## SUPPLEMENTARY DATA

**Files in this Data Supplement:**

- SUPPLEMENTARY DATA
