# Supplementary material for: Protein kinase C controls activation of the DNA integrity checkpoint
Source: Nucleic Acids Res. 2014 May 3;42(11):7084–95. doi: 10.1093/nar/gku373 (PMC4066786; doi:10.1093/nar/gku373)
Supplement: SUPPLEMENTARY DATA [file supp_gku373_nar-03041-d-2013-File010.pdf]

SUPPLEMENTARY DATA

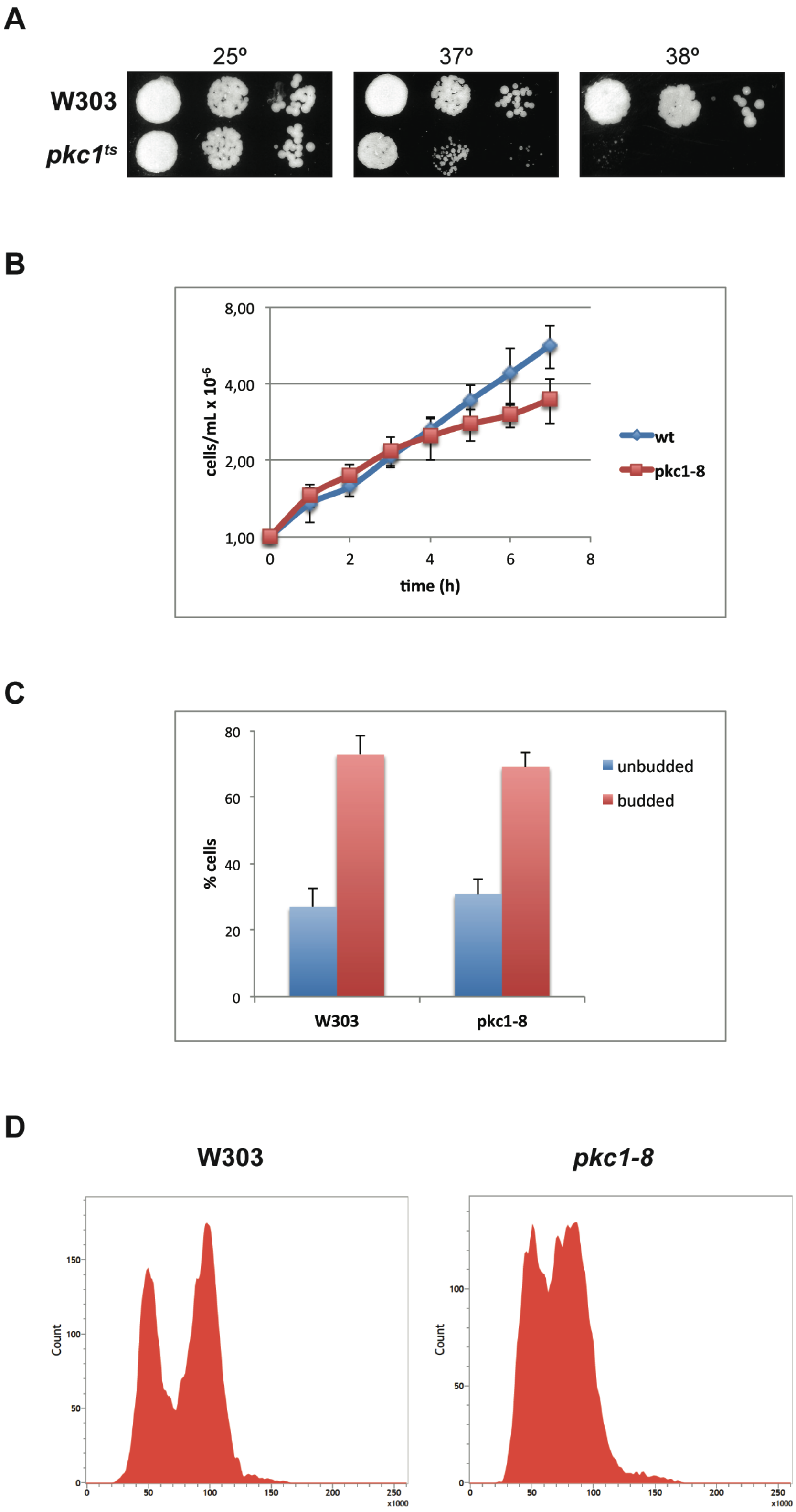

Supplementary Figure 1. Analysis of the thermosensitive growth defect of the *pkc1*^{ts} strain. A) 10-fold serial dilutions from exponentially growing cultures of the *pkc1*^{ts} (JC6-3a) strain were spotted onto YPD medium and plates were incubated at 25°, 37º or 38º for 3 days. B) Exponentially growing cultures of the wild type (W303) and *pkc1*^{ts} (JC6-3a) strains were transferred at 37º and the number of cells were determined in a Z2 Coulter Counter at the indicated times. C) Budding index of wild type and *pkc1*^{ts} samples after 4 h incubation at 37º. D) Analysis of DNA content by flow cytometry in wild type and *pkc1*^{ts} cells after 4 h incubation at 37º.

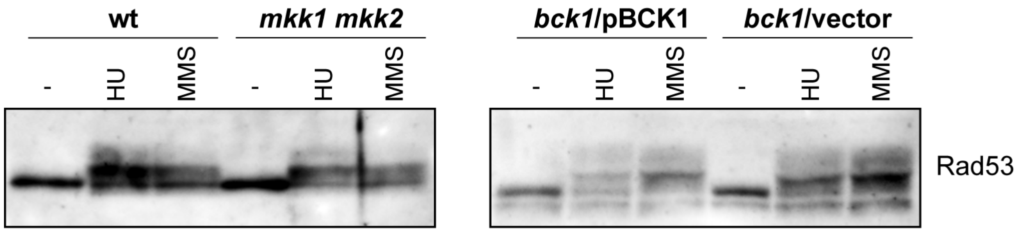

Supplementary Figure 2. Analysis of Rad53 checkpoint kinase activation in mutant strains in Pkc1-regulated MAP kinase cascade. A) Exponentially growing cultures of the wild type (W303-1a), *mkk1 mkk2* and *bck1* strain transformed with an empty vector or a centromeric plasmid containing de *BCK1* gene, were split and incubated for one hour in the absence or presence of 200 mM hydroxyurea or 0.04% MMS. Activation of the checkpoint kinase Rad53 was determined by western analysis as slower migrating bands.

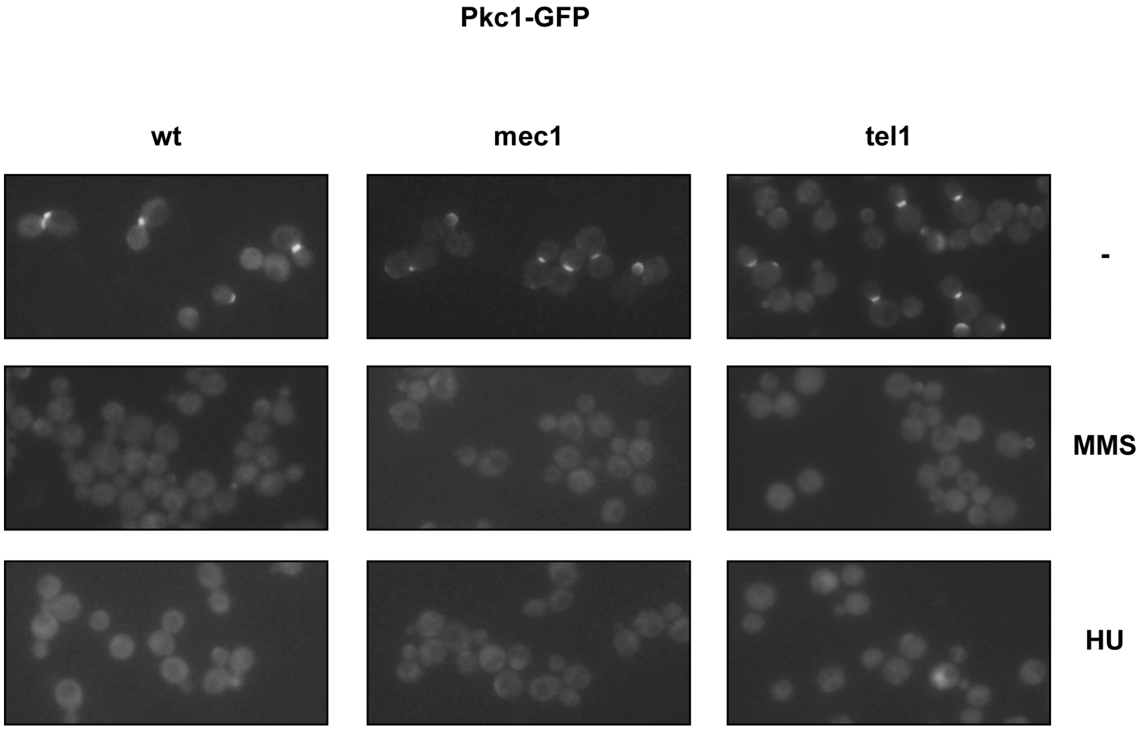

Supplementary Figure 3. Analysis of Pkc1 subcellular localization after genotoxic stress in checkpoint mutant strains. Exponentially growing wild type, *mec1sml1* and *tel1* cells expressing a GFP-tagged Pkc1 protein were incubated for 1.5 hour in the absence or presence of 0.2 M hydroxyurea or 0.04% MMS. GFP fluorescence signal are shown.

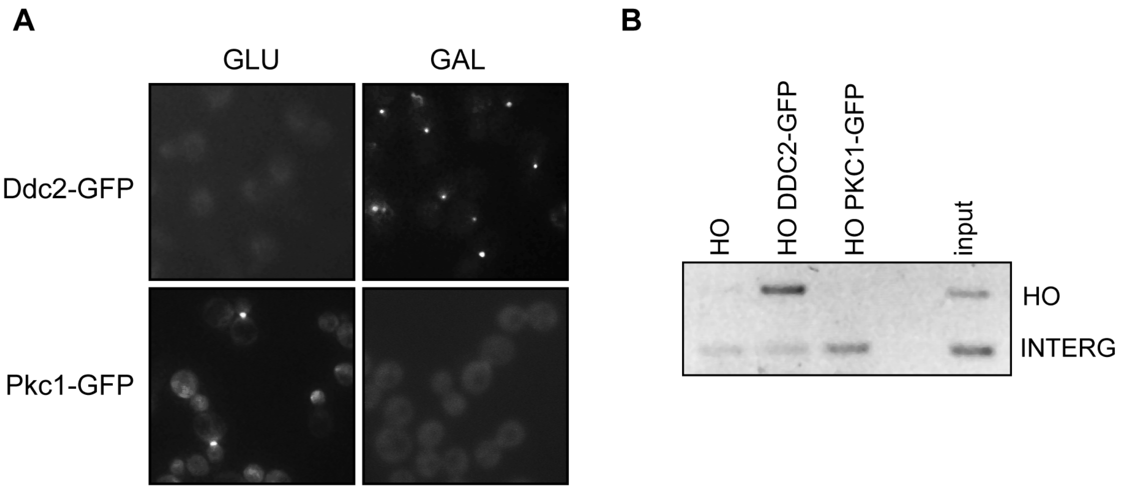

Supplementary Figure 4. Analysis of Pkc1 binding to the site of DNA damage. Raffinose-grown CCG2781 (*MATa ade1-100 trp1-1 leu2-3,112 lys5 ura3-52 trp1::hisG hoΔ hml::ADE1 hmr::ADE1 ade3::GAL1-HO DDC2-GFP-kanMX4*) and JCY1514 (*MATa ade1-100 trp1-1 leu2-3,112 lys5 ura3-52 trp1::hisG hoΔ hml::ADE1 hmr::ADE1 ade3::GAL1-HO PKC1-GFP-kanMX4*) cells were incubated for 6 hours in the presence of 2% glucose or 2% galactose in order to induce a DSB at the HO cleavage site. A) Binding of Ddc2 and Pkc1 proteins to the site of damage was analyzed by the appearance of fluorescence foci. B) Binding of Ddc2 and Pkc1 proteins to the site of damage was analyzed by chromatin immunoprecipitation assay. Pkc1 and Ddc2 were purified by immunoprecipitation with an anti GFP antibody and the co-purification of a DNA fragment around the HO cleavage site and an independent intergenic region in the immunoprecipitates was tested by PCR. Control PCRs of the sample from the non-tagged strain and the input genomic DNA are shown.

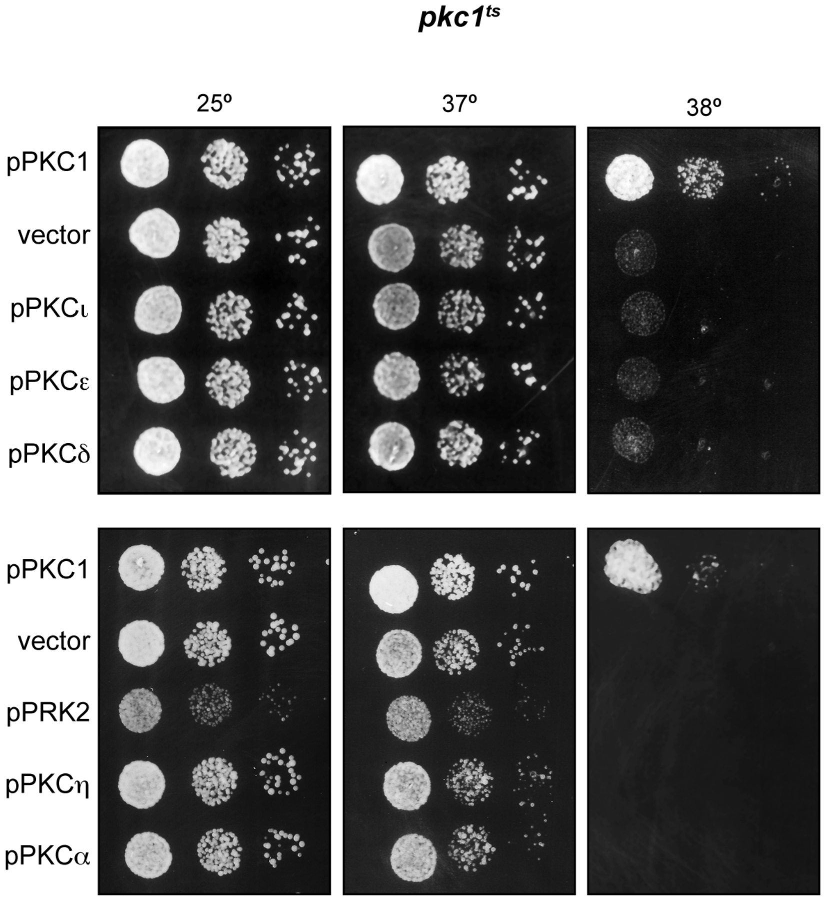

Supplementary Figure 5. Growth analysis of the *pkc1*^{ts} mutant cells expressing mammalian PKC isoforms. 10-fold serial dilutions from exponentially growing cultures of the *pkc1*^{ts} (JC6-3a) strain transformed with a centromeric plasmid containing yeast *PKC1* or mammalian PKCα, PKCδ, PKCε, PKCι, PKCη or PRK2 genes (expressed under the control of *PKC1* promoter) were spotted onto YPD medium and plates were incubated at 25°, 37° or 38° for 3 days.

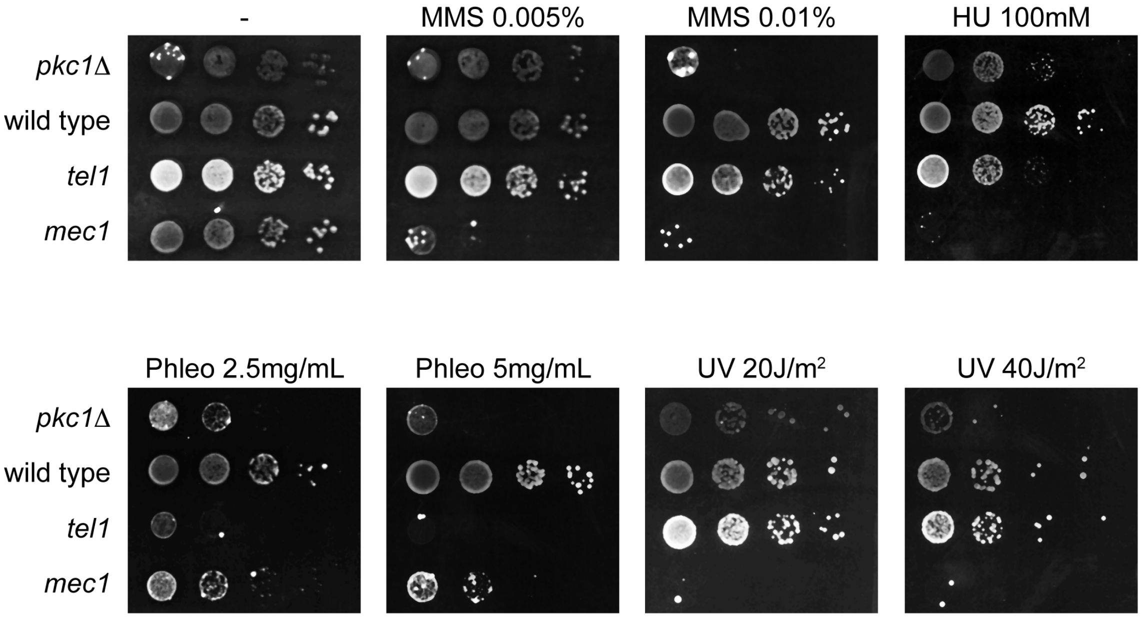

Supplementary Figure 6. Hypersensitivity of *pkc1* and checkpoint mutants to genotoxic stress. 10-fold serial dilutions from exponentially growing cultures of the wild type, *pkc1Δ*, *tel1* and *mec1-1* strains were spotted onto YPD 1M sorbitol medium containing different amounts of genotoxic drugs or exposed to UV radiation as indicated. Plates were incubated at 25°for 3 days.

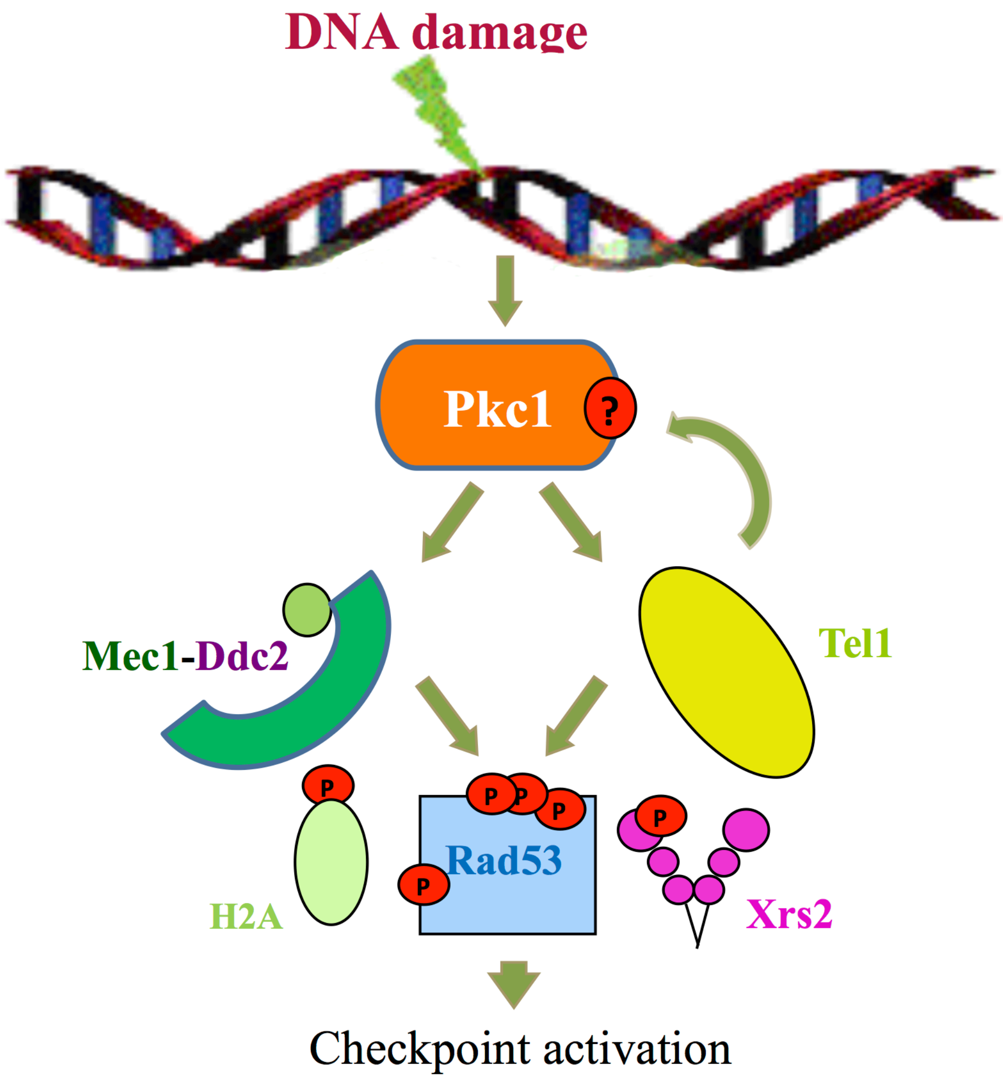

Supplementary Figure 7. Model of the regulation of the DNA integrity checkpoint by Pkc1.
